# Preservation of Intrinsically Photosensitive Retinal Ganglion Cells (ipRGCs) in Late Adult Mice: Implications as a Potential Biomarker for Early Onset Ocular Degenerative Diseases

**DOI:** 10.1167/iovs.65.1.28

**Published:** 2024-01-15

**Authors:** Anna Matynia, Brandy S. Recio, Zachary Myers, Sachin Parikh, Rajesh Kumar Goit, Nicholas C. Brecha, Luis Pérez de Sevilla Müller

**Affiliations:** 1 Department of Ophthalmology, Jules Stein Eye Institute, David Geffen School of Medicine, University of California, Los Angeles, Los Angeles, California, United States; 2 Brain Research Institute, University of California, Los Angeles, Los Angeles, California, United States; 3 Department of Neurobiology, David Geffen School of Medicine, University of California, Los Angeles, Los Angeles, California, United States; 4 VA Greater Los Angeles Healthcare System, Los Angeles, California, United States; 5 Department of Medicine, David Geffen School of Medicine, University of California, Los Angeles, Los Angeles, California, United States

**Keywords:** melanopsin, aging, mouse retina, function, biomarker

## Abstract

**Purpose:**

Intrinsically photosensitive retinal ganglion cells (ipRGCs) play a crucial role in non–image-forming visual functions. Given their significant loss observed in various ocular degenerative diseases at early stages, this study aimed to assess changes in both the morphology and associated behavioral functions of ipRGCs in mice between 6 (mature) and 12 (late adult) months old. The findings contribute to understanding the preservation of ipRGCs in late adults and their potential as a biomarker for early ocular degenerative diseases.

**Methods:**

Female and male C57BL/6J mice were used to assess the behavioral consequences of aging to mature and old adults, including pupillary light reflex, light aversion, visual acuity, and contrast sensitivity. Immunohistochemistry on retinal wholemounts from these mice was then conducted to evaluate ipRGC dendritic morphology in the ganglion cell layer (GCL) and inner nuclear layer (INL).

**Results:**

Morphological analysis showed that ipRGC dendritic field complexity was remarkably stable through 12 months old of age. Similarly, the pupillary light reflex, visual acuity, and contrast sensitivity were stable in mature and old adults. Although alterations were observed in ipRGC-independent light aversion distinct from the pupillary light reflex, aged wild-type mice continuously showed enhanced light aversion with dilation. No effect of sex was observed in any tests.

**Conclusions:**

The preservation of both ipRGC morphology and function highlights the potential of ipRGC-mediated function as a valuable biomarker for ocular diseases characterized by early ipRGC loss. The consistent stability of ipRGCs in mature and old adult mice suggests that detected changes in ipRGC-mediated functions could serve as early indicators or diagnostic tools for early-onset conditions such as Alzheimer's disease, Parkinson's disease, and diabetes, where ipRGC loss has been documented.

Visual function declines with age,^1^ with aging affecting both the cornea and lens required for a clear light path, photoreceptors and their circuitry, and visual processing in the brain. Manifestations of normal aging include decreased visual acuity, contrast sensitivity and the onset of sleep and circadian dysfunction,^2^^,^^3^ which are linked to both rod and cone photoreceptors and intrinsically photosensitive retinal ganglion cells (ipRGCs) in the retina.^4^^–^^10^

ipRGCs containing the photopigment melanopsin^8^^–^^10^ are well conserved across species, including humans,^11^^–^^14^ and are located in the retinal ganglion cell layer (GCL), with a large displaced population in the inner nuclear layer (INL) in humans and a small displaced population in mice.^15^ ipRGCs are a class of photoreceptors that mediate both non–image-forming functions of the eye^16^ and vision-forming pathways. ipRGC functions include photoentrainment of circadian rhythms, modulation of the sleep/wake cycle, masking response, sleep regulation, control of pupillary light reflex, light-induced suppression of melatonin secretion, mood regulation,^17^^,^^18^ and color pathway and brightness perception.^19^^–^^23^

With age, a number of extraretinal changes can alter visual function. For example, age-related changes in lens clarity and density^24^^,^^25^ can reduce the transmission of blue light, which is known to suppress ipRGC-mediated melatonin secretion during the day.^26^ A further age-related change in the eye that may contribute to reduced levels of light reaching the ipRGCs is the reduction in pupil size.^27^ As a result, this diminished blue light input to the circadian clock has been shown to result in disturbed circadian rhythm and sleep in the elderly.^2^^,^^7^^,^^28^^,^^29^ In addition to these non-specific decreases in retinal illumination, ipRGCs are often disrupted in many neurodegenerative disorders including Alzheimer's disease (AD),^30^^–^^33^ Parkinson's disease (PD),^34^^–^^37^ Huntington's disease,^38^^,^^39^ glaucoma,^40^^–^^42^ and diabetes,^43^^,^^44^ suggesting that ipRGC-mediated functions may be useful biomarkers of early onset disease. Therefore, it is crucial to study the effects of normal aging in mature and old adult control animals to facilitate identification of functional and anatomical retinal impairments resulting from early disease onset from younger adults. Given the reported correlation of ipRGC pathologies with ophthalmic diseases, there is a potential of ipRGCs to serve as biomarkers in many neurodegenerative diseases.

In this study, we present a comparison of ipRGC morphology in mature adult (6 months old) and old adult (12 months old) mice and its correlation with behavioral functions mediated by the melanopsin system. In addition, sex-related differences in most neurodegenerative diseases are increasingly recognized, particularly in early-onset conditions such as PD, that occur before the age of 40 or 50 years,^45^^,^^46^ as well as early-onset AD, which affects individuals in their 30s and 40s.^47^^–^^49^ Men tend to be more commonly affected by early-onset PD, possibly due to hormonal and genetic factors.^50^^,^^51^ Conversely, early-onset AD exhibits a higher prevalence in women, potentially influenced by sex-specific genetic variants and hormonal fluctuations throughout their reproductive life.^52^^,^^53^ Moreover, sex-related disparities are evident in nonarteritic anterior ischemic optic neuropathy occurring in the late 30s^54^ and diabetic retinopathy, which can manifest as early as the age of 30.^55^ The underlying mechanisms for these differences involve complex interactions among sex hormones, genetic predispositions, and immune responses. Recognizing and understanding these sex-specific aspects of neurodegenerative and ocular diseases are vital for developing tailored treatments and personalized approaches that consider the distinct needs of male and female patients, ultimately leading to improved therapeutic outcomes and enhanced quality of life. Therefore, we have included an analysis of the differences between male and female mice in ipRGC function and M1 ipRGC subtype morphology.

## Materials and Methods

These studies were conducted under protocols approved by the University of California at Los Angeles (UCLA) Animal Research Committee. All experiments were carried out in accordance with guidelines for the welfare of experimental animals issued by the U.S. Public Health Service Policy on Humane Care and Use of Laboratory Animals, and the UCLA Animal Research Committee.

### Animals

Female and male C57BL/6J mice (000664, bred at UCLA; The Jackson Laboratory, Bar Harbor, ME, USA) at 6 and 12 months of age (a total of 39 animals) were used in this study, and data analyzed from past experiments represented an additional 190 animals. All mice were housed in standard cages in a temperature-controlled room on a 12-hour light/dark cycle with free access to standard pellet food and water. Opn4^DTA^ mice (035927, Opn4tm3.1(DTA)Saha/J; The Jackson Laboratory) express the diphtheria toxin subunit alpha (DTA) sequence under the control of the Opn4 promoter (exons 1–9 of the opsin 4), resulting in ablation of the ipRGCs.^56^ These mice were backcrossed into the C57Bl/6J background and bred heterozygous to heterozygous, with ipRGC ablation verified using a qualitative pupillary light reflex assay as reported previously.^57^

### Behavioral Assays

The light-aversion assay was performed as previously described with modifications.^57^^,^^58^ A two-chamber box with open, light and closed, dark sections was used to measure time spent in the light compartment. An overhead LED lighting system, with adjustable illumination from 0 to 1000 lux calibrated with a light meter (HHLM-2; Omega Engineering, Norwalk, CT, USA), a standard LED spectrum, and diffusers provided uniform illumination in the open, lit side of the chamber. Behavior was monitored using an infrared light source and video camera with white light filter, and automated tracking and analysis were performed with a video tracker (Med Associates, St. Albans, VT, USA) and an activity monitor (Med Associates), respectively. Mice were acclimated to a dimly lit room (less that 10 lux) for at least 15 minutes and dark adapted prior to testing for at least 10 minutes. Light aversion was tested at 0 and 1000 lux: the 0-lux test was used as baseline to calculate aversion indices. A 1% Atropine Sulfate Ophthalmic Solution (Akorn, Lake Forest, IL, USA) was used as a dilating agent where indicated.

### Pupillary Light Reflex

Unanesthetized mice were used for pupillometry as previously described.^57^ Before each experiment, mice were acclimated in a dimly lit room (less than 50 lux) for at least 10 minutes. The pupillary light reflex (PLR) was measured with a hand-held slit lamp (Kowa SL-15; Kowa Pharmaceuticals, Montgomery, AL, USA) using a semiquantitative scale, where 1 *=* pinpoint pupil and 3 *=* no constriction.

### Visual Acuity and Contrast Sensitivity

Mice were acclimated in a dimly lit room (less than 50 lux) for at least 20 minutes prior to testing in a virtual automated optomotor system (OptoDrum; Striatech, Clearwater, FL, USA).^59^^–^^63^ Briefly, a mouse was placed on the center platform of an enclosed chamber with four computer screens as walls that presented stripes of varying thickness and contrast. Head movement was captured by an overhead camera and tracking was computationally determined. Mice were tested for visual acuity using 99.7% contrast with spatial resolution between 0.056 and 0.50 cycles per degree. Contrast sensitivity was assessed at multiple spatial resolutions with contrast between 0.9% and 99.7%, using the reciprocal of Michelson's contrast threshold. Light intensity at the mouse's cornea was in the mesopic range at approximately 90 to 110 lux.

### Immunohistochemistry in Whole-Mounted Retinas

Following deep anesthesia with 1% to 3% isoflurane (Abbott Laboratories, Abbott Park, IL, USA), animals were euthanized by cervical dislocation or decapitation. The eyes were enucleated and dissected in Hibernate A (Invitrogen, Carlsbad, CA, USA) for fluorescence and immunohistochemical studies. The retinas were removed from the eyecups, and four small incisions were made on each retina to lay the tissue flat. Retinas were mounted onto nitrocellulose membrane filters (EMD Millipore, Billerica, MA, USA), with the GCL facing upward, and fixed for 15 minutes in 4% paraformaldehyde in 0.1-M phosphate buffer (PB) at room temperature.

Immunohistochemical labeling was performed using our published protocols.^64^^–^^67^ After fixation, the whole-mounted retinas were subsequently washed in PB three times for a total of 90 minutes and incubated in 10% normal goat serum with 0.5% Triton X-100 at 4°C overnight. Retinas were removed from the blocking solution and subsequently incubated in anti-melanopsin primary antibodies (1:1000, AB-N39; Advanced Targeting Systems, San Diego, CA, USA) for 7 days at 4°C. They were then rinsed three times for 30 minutes each with 0.1-M PB and incubated with the corresponding secondary antibodies (1:1000, Goat anti-Rabbit IgG Alexa Fluor 488 or Alexa Fluor 594; Invitrogen) overnight at 4°C. The following day, the retinas were washed three times in 0.1-M PB for a total of 90 minutes and subsequently placed on a microscope slide with the GCL facing upward. Then, the samples were mounted in Aqua-Poly/Mount (Polysciences, Warrington, PA, USA), and the coverslips were sealed with nail polish. As a negative control, the omission of the primary antibody confirmed the elimination of specific labeling.

### Fluorescent Image Acquisition

Labeling was assessed with a Zeiss laser scanning microscope (Zeiss LSM 880; Carl Zeiss Microscopy, Jena, Germany) with a Zeiss C-Apochromat 40×/1.2 corrected water immersion objective. The images were captured at a resolution of 1024 × 1024 pixels.

### M1 ipRGC Morphological Analysis

M1 ipRGCs from all retinal quadrants (nasal, temporal, superior, and inferior) were reconstructed using Imaris 9.5.0 (Bitplane AG, Concord, MA, USA) with the Filament Tracer option. The Filament Tracer operates on three-dimensional images, which provides sufficient resolution to resolve the filaments to be studied in all three spatial directions. The Filament Tracer option automatically computes all the paths from a user-defined starting point (ipRGC body) to the end of the structure. The filaments were then manually traced by the user. Imaris provided the dendrite length, Sholl analysis, and total number of dendritic branch points. M1 densities and soma sizes were measured with Imaris software.

### Statistics

All values are given as mean and standard error of the mean (SEM). Single statistical comparisons of a group versus its control group were performed using a two-tailed Student's *t*-test or two-way ANOVA as indicated in Prism 4.0 or 9.0 (GraphPad, Boston, MA, USA). If data were not normally distributed, non-parametric tests (Mann–Whitney *U* test) were used. *P* ≤ 0.05 was considered statistically significant.

## Results

### Behavioral Consequences on Light Aversion and Pupil Dilation in Mice

**Figure 1.**
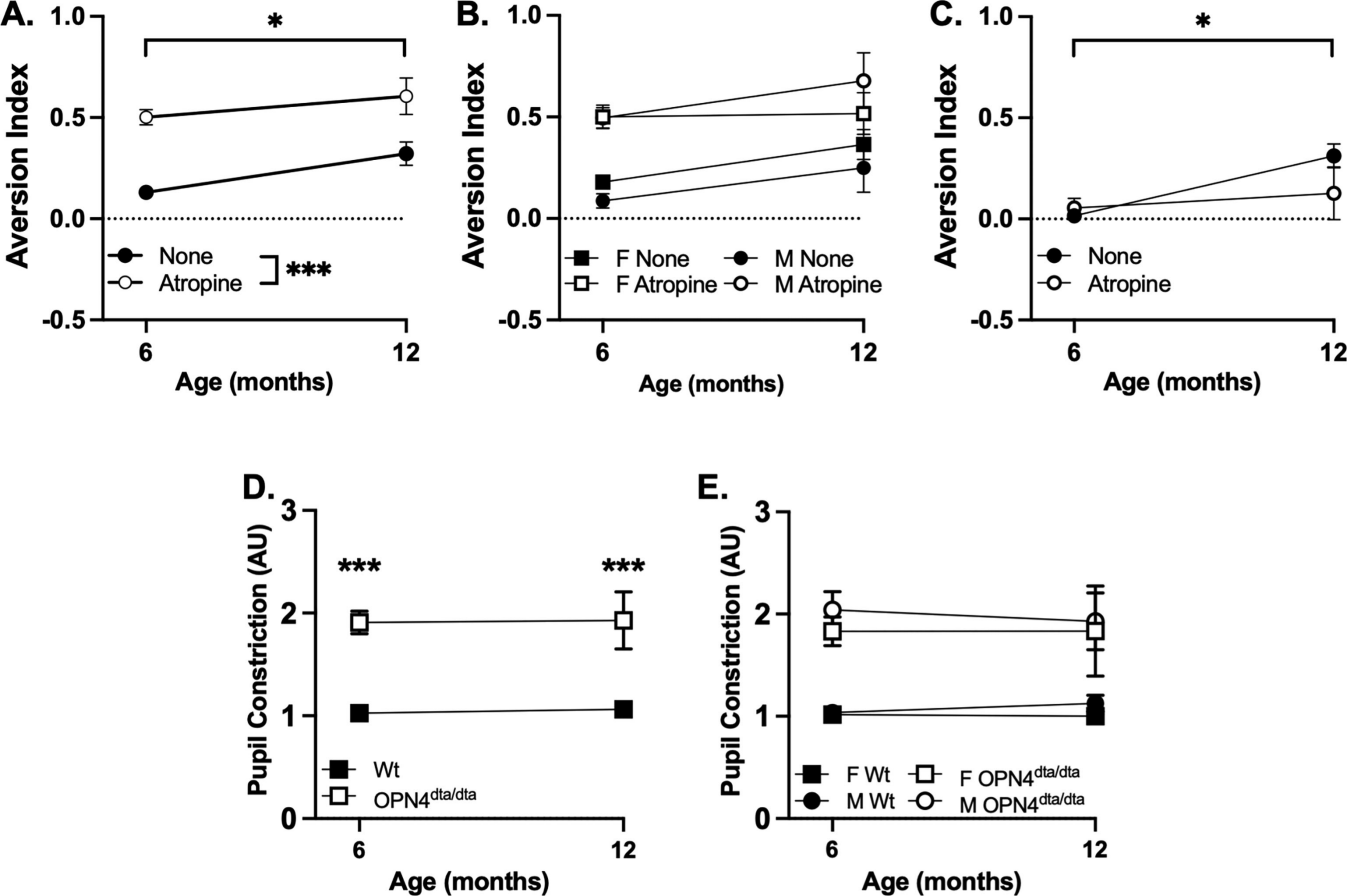
Light aversion and pupillary light reflex in mature and old adult mice. (**A**) Light aversion tested at 1000 lux with and without atropine eye drops in 6- and 12-month-old wild-type mice. (**B**) Light aversion tested at 1000 lux with and without atropine eye drops in 6- and 12-month-old wild-type mice by sex. (**C**) Light aversion tested at 1000 lux with and without atropine eye drops in 6- and 12-month-old mice lacking melanopsin-expressing neurons. (**D**) Pupillary light reflex in 6- and 12-month-old wild-type mice and mice lacking melanopsin-expressing neurons. (**E**) Pupillary light reflex in 6- and 12-month-old wild-type mice and mice lacking melanopsin-expressing neurons by sex. For light aversion in wild-type mice, there was a significant main effect of age (*P =* 0.03; *F*_1,166_ *=* 5.02) and of eye drops (*P* < 0.0001; *F*_1,166_ *=* 24.68), but no interaction (*P =* 0.50; *F*_1,166_ *=* 0.45). No effect of sex was observed. For light aversion in the OPN4^dta/dta^ mice, there was a significant main effect of age (*P =* 0.01; *F*_1,76_ *=* 6.38) but not of eye drops (*P =* 0.32; *F*_1,76_ *=* 1.01) and no interaction (*P =* 0.13; *F*_1,76_ *=* 2.39). No effect of sex was observed. For pupillometry in wild-type mice, there was a significant main effect of genotype (*P <* 0.00001; *F*_1,90_ *=* 84.58) but not of age (*P =* 0.91; *F*_1,24_ *=* 0.011) and no interaction (*P =* 0.76; *F*_1,24_ *=* 0.10). No effect of sex was observed.

To investigate whether aging results in detectable functional deficits in ipRGCs in mice, we tested light aversion behavior with and without pupil dilation (Fig. 1). Wild-type mice exhibited increased light aversion with atropine dilation for both sexes (6-month-old males, *n* = 41; 12-month-old males, *n* = 3; 6-month-old females, *n* = 32; 12-month-old females, *n* = 6) (Figs. 1A, 1B). Mice lacking ipRGCs (6-month-old males, *n* = 20; 12-month-old males, *n* = 1; 6-month-old females, *n* = 14; 12-month-old females, *n* = 5) exhibited decreased light aversion that did not increase with dilation (Fig. 1C), consistent with previous reports.^57^^,^^68^^,^^69^ For both wild-type and mice lacking ipRGCs, there was an increase in light aversion without dilation at 12 months old compared to 6 months old and for wild-type mice with dilation (Figs. 1A, 1C). No age-dependent effect was observed in mice lacking ipRGCs with atropine dilation. There were no significant differences in pupil constriction to bright light in 6-month-old and 12-month-old wild-type mice or mice lacking ipRGCs for either sex (Figs. 1E, 1F). No effect of sex was observed in any of these metrics. As previously reported, mice lacking ipRGCs have significantly reduced pupil constriction compared to wild-type mice.^57^^,^^70^^,^^71^

**Figure 2.**
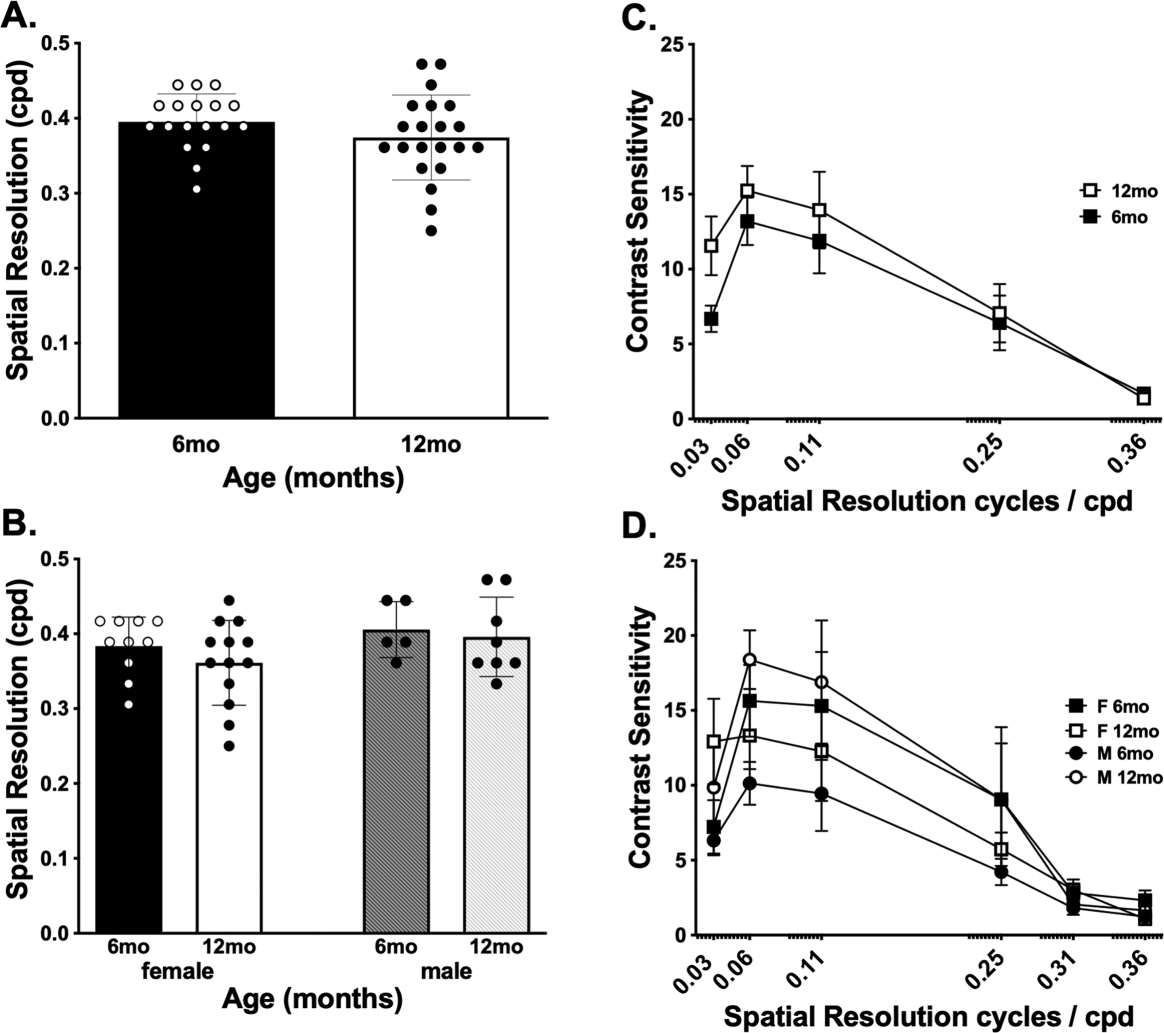
Visual acuity and contrast sensitivity in mature and old adult mice. (**A**) Spatial resolution in 6- and 12-month-old mice. (**B**) Spatial resolution in 6- and 12-month-old mice by sex. (**C**) Contrast sensitivity function in 6- and 12-month-old mice. (**D**) Contrast sensitivity function in 6- and 12-month-old mice by sex. No significant differences were found in visual acuity with age or sex using Student's *t*-test. There was a significant main effect of contrast (*P* > 0.0001; *F*_5,136_ *=* 23.3), but no main effect of age (*P =* 0.10; *F*_1,136_ *=* 2.7), and no interaction (*P =* 0.72; *F*_5,136_ *=* 0.57).

ipRGCs have been shown to contribute to both visual acuity and contrast sensitivity. The M4 class of ipRGCs responds to contrast gradients by electrophysiological recordings, and functional deficits have been observed using an optomotor task.^56^^,^^72^ To determine if mature adulthood impacts these functions, wild-type mice were tested at 6 months old or 12 months old (*n* = 13 males, *n* = 23 females) (Fig. 2B). Visual acuity remained stable even though more variability occurred in the older mice. Similarly, contrast sensitivity was not affected. However, a trend toward increased contrast sensitivity at lower spatial resolution in older adult mice was observed (Figs. 2C, 2D).

### Morphological Analysis of M1 Cells

**Figure 3.**
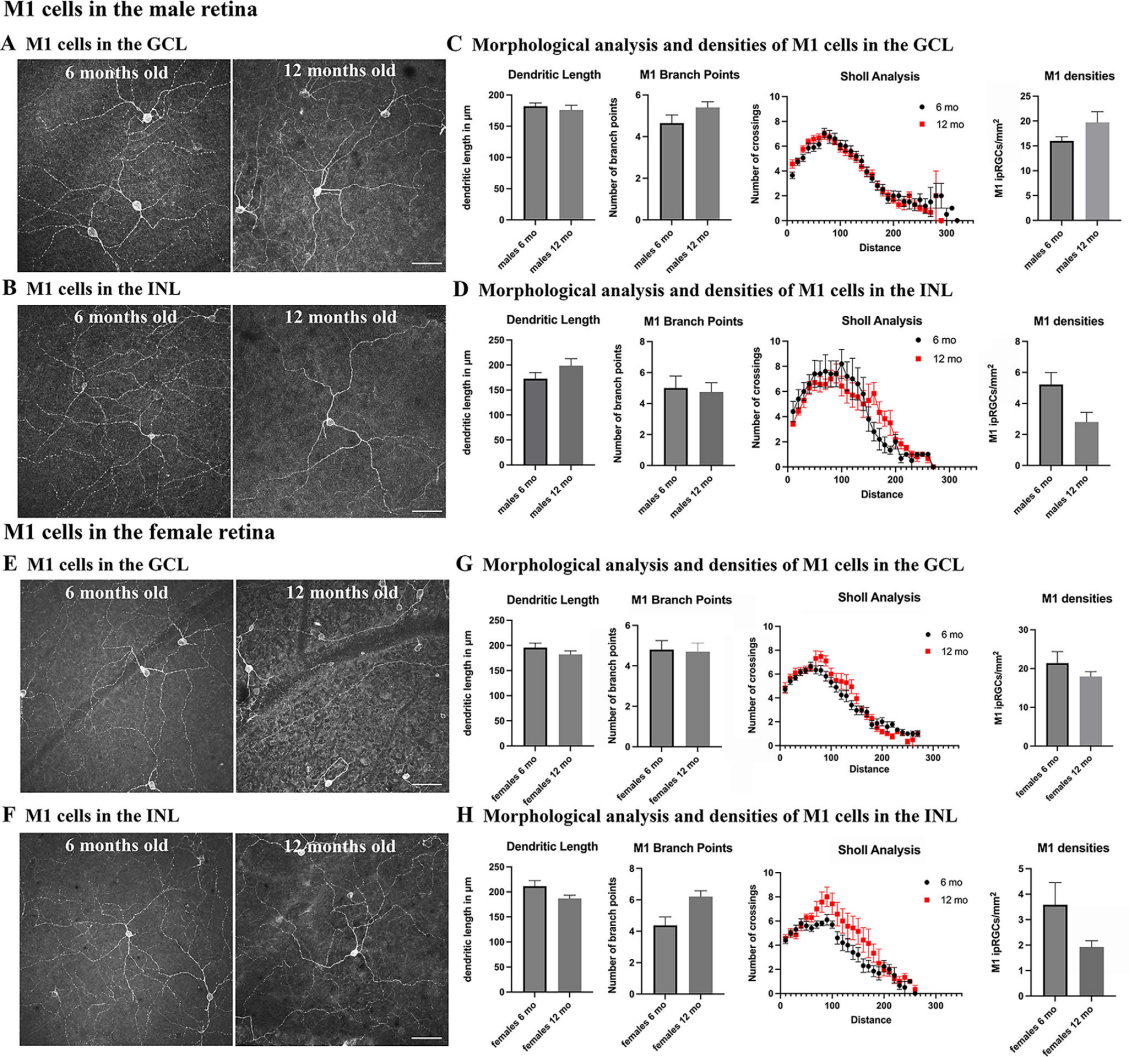
Dendritic structure and densities of M1 cells in male and female retinas in the GCL and INL. (**A**) Melanopsin staining in 6- and 12-month-old male wild-type retinas in the GCL. (**B**) Melanopsin staining in 6- and 12-month-old male wild-type retinas in the INL. (**C**) Quantification of morphological parameters examined for M1 cells at 6 and 12 months of age in the GCL. No significant differences were found in dendritic length, number of branch points, number of crossings in a Sholl analysis, or density. (**D**) Quantification of morphological parameters examined for M1 cells in 6- and 12-month-old retinas in the INL. No significant differences were found in dendritic length, number of branch points, number of crossings in a Sholl analysis, or density. (*P <* 0.05). (**E**) Melanopsin staining in 6- and 12-month-old female wild-type retinas in the GCL. (**F**) Melanopsin staining in 6- and 12-month-old female wild-type retinas in the INL. (**G**) Quantification of morphological parameters examined for M1 cells in 6- and 12-month-old retinas in the GCL. No significant differences were found in dendritic length, number of branch points, number of crossings in a Sholl analysis, or density. (**H**) Quantification of morphological parameters examined for M1 cells in 6- and 12-month-old retinas in the INL. No significant differences were found in dendritic length, number of branch points, number of crossings in a Sholl analysis, or density (*P <* 0.05). *Scale bar*: 50 µm (**A**, **B**, **E**, **F**).

To investigate whether morphological changes occurred in ipRGCs in the GCL and INL in male and female retinas, we used an antibody that stains different types of ipRGCs.^73^ We focused on the M1 ipRGCs, as they are the main type of ipRGCs with a high content of melanopsin. They are easily differentiated from other ipRGC types in the GCL due to their high content of melanopsin immunoreactivity and two to five primary dendrites that stratify in one single layer at the outmost part of the IPL as previously reported^73^^,^^74^ (Figs. 3A, 3E). ipRGCs located in the INL are exclusively M1 cells^73^^,^^74^ (Figs. 3B, 3F).

Soma sizes were evaluated in male mice 6 months and 12 months old, as well as in female mice of corresponding age groups. In the male 6-month-old group, soma sizes ranged from 11 to 18 µm, with an average of 13.4 ± 2.2 µm (*n =* 83 cells from three retinas from three male mice). Similarly, for male mice 12 months old, soma sizes ranged from 9 to 16 µm, with an average of 12.5 ± 1.6 µm (*n =* 98 cells from three retinas from three male mice). Among the female mice at 6 months, soma sizes ranged from 9 to 17 µm, with an average of 12.9 ± 1.9 µm (*n =* 112 cells from three retinas from three female mice). In 12-month-old females, soma sizes ranged from 9 to 17 µm, with an average of 12.2 ± 1.9 µm (*n =* 85 cells from three retinas from three female mice). No statistically significant differences were observed in soma sizes across the distinct age and sex groups. Morphological analysis of M1 ipRGCs was performed using Imaris 9.5.0, and information on dendritic length, total number of dendrite branch points, and Sholl analysis were determined (Figs. 3C, 3D, 3G, 3H).

Our data showed that M1 cells in the GCL and INL did not exhibit significant morphological differences in their dendritic complexity between 6 and 12 months of age in either males (*n =* 19 cells in the GCL and *n =* 5 cells in the INL from five retinas from three male mice at 6 months old; *n =* 28 cells in the GCL and *n =* 8 cells in the INL from four retinas from four male mice at 12 months old) (Figs. 3A–3D) or females (*n =* 27 cells in the GCL and *n =* 10 cells in the INL from four retinas from four female mice at 6 months old; *n =* 29 cells in the GCL and *n =* 7 cells in the INL from eight retinas from six female mice at 12 months old) (Figs. 3E–3H). There were no significant differences observed between male and female mice in any of these parameters. In both the 6-month-old and 12-month-old groups, the density of M1 cells per square millimeter exhibited no discernible disparity between males (*n =* 5 retinas at 6 months old; *n =* 4 retinas at 12 months old) and females (*n =* 3 retinas at 6 months old; *n =* 4 retinas at 12 months old) (Figs. 3C, 3D, 3G, 3H). Furthermore, no significant differences were detected within either age group or between genders. Although a decrease in M1 cell density was observed in both female and male retinas in the INL (Figs. 3D, 3H), this difference was not found to be statistically significant. These findings suggest that, in this study, age and sex did not have a significant impact on the measured structural characteristics of M1 neurons in the mice.

## Discussion

Aging is associated with visual dysfunction, including reduced sensitivity of the circadian system to light, altered timing of circadian rhythms relative to nocturnal sleep, and increased sleep disturbances.^75^^–^^77^ These functions are in part mediated by ipRGCs, although there are differences in the literature regarding RGC loss during aging, which may vary depending on the species and model studied. Although loss and/or morphological changes in RGCs have been observed in aged rodents and human,^78^^–^^80^ other groups have not observed neuronal loss in the GCL in aged rats.^81^^–^^83^

Interestingly, human retinas show a relatively stable density of ipRGCs over time that is maintained in healthy subjects until the age of 70,^84^ after which there is a decline of ipRGC density and atrophy of the dendritic arborizations in all ipRGC types.^84^ This could explain the circadian rhythm desynchronization in the elderly.^3^^,^^85^^–^^88^ Studies have also reported that ipRGC density and morphology are maintained in normal rats at 12 and 18 months of age,^81^^,^^89^^,^^90^ which is consistent with our study in mice. However, the discrepancy between these studies could be attributed to the use of different animal models and their genetic backgrounds. For example, in Sprague Dawley rats, ipRGCs showed no significant morphological changes associated with age, but the mean density of ipRGCs in P23H rats showed a 67% decrease between 4 and 18 months of age.^89^ Additionally, in 2-years-old rodless and coneless mice (*rd/rd cl*), the retinas showed normal levels of melanopsin expression, and immunocytochemistry assays demonstrated a maintained morphology of ipRGCs.^91^

### ipRGC Function With Age

Retinal function is known to be affected with age,^92^^,^^93^ and this can vary depending on biological sex, with female Sprague Dawley rats exhibiting better preserved retinal function at 18 months compared to males.^94^ Aging also impacts circadian rhythms,^2^^,^^7^^,^^28^^,^^29^ partly due to altered function of the ipRGC types. With aging, corneas and lenses undergo changes that result in less blue light reaching the retina, leading to reduced activation of blue light–sensitive ipRGCs.^26^^,^^27^ As a result, it could be speculated that ipRGC-dependent functions such as pupil response to light, light aversion, visual acuity, and contrast sensitivity may be reduced in older individuals.^95^^–^^98^ However, ipRGC-mediated circadian and pupillary responses to light are maintained in the absence of rods and cones,^91^ and no age-related changes in pupil responses are found in humans.^99^^,^^100^ These findings are also consistent with a study in which the magnitude of sustained pupillary constriction responses to blue-light and green-light stimuli did not exhibit significant changes between young and older human subjects,^101^ as well as our studies in mice presented here.

Although the PLR responses of the Royal College of Surgeons (RCS) rats at 12 months of age were diminished compared to those of normal, non-dystrophic rats,^102^ it seems that there are some age-dependent compensatory mechanisms to preserve PLR^99^^,^^102^ and even to enhance pupil responses mediated by ipRGC in humans.^99^ The improvement of the PLR at older ages may reflect some compensatory mechanisms in the inner retina, as well as in the central connections of the PLR pathway, to preserve the PLR responses. Taken together, the robustness of the PLR to aging indicates a highly conserved and reliable mechanism that may reflect more than ipRGC function alone.

ipRGCs also mediate light aversion, which is clinically observed in conditions such as migraine as heightened sensitivity to light, known as photoallodynia or photophobia, and is defined as light-enhanced or -induced pain. In both humans and mice, the spectral properties of photoallodynia implicate ipRGCs,^103^^–^^108^ a suggestion that was confirmed using mice lacking ipRGCs without pathophysiology and in specific disease models.^57^^,^^58^^,^^109^ In migraine, rods, cones and ipRGCs^58^ were shown to be equally essential in a rodent model, with ipRGCs selectively involved in photophobia between migraine episodes.^110^ However, a role for green cone photoreceptors for amelioration of migraine symptoms in humans was also indicated.^111^ In neonatal mice, photoaversion has been further mapped to a specific M1 class of ipRGCs,^112^ and a specific photoreceptor pathway for photophobia in *Drosophila* larvae has been identified, which, due to their projection regions controlling circadian photoentrainment, is likely functionally related to ipRGCs.^113^ ipRGCs persist during normal aging but are susceptible to degeneration even in mature adults in AD, PD, glaucoma, and diabetic retinopathy.^114^ Our data indicate that ipRGCs are functionally maintained in light aversion in mice up to 12 months old, regardless of whether they are localized to the GCL or are displaced in the INL. Taken together, the role of ipRGCs in specific pathophysiologies is evident, and the loss of light aversion in mice lacking ipRGCs makes this differential a potential biomarker for ipRGC degeneration.

Visual acuity, a measure of sharpness and clarity of vision, diminishes with age and is a major health issue. In humans, the most common causes of reduced visual acuity are glaucoma and macular degeneration, including age-related macular degeneration and myopia-induced macular degeneration.^115^^–^^118^ Similar age-related loss of visual acuity has been observed in rodent models, although loss of visual acuity usually occurs by around 1.5 to 2 years of age, consistent with our results indicating normal visual acuity in mice at 12 months old. Dysfunction or degeneration most frequently occurs in photoreceptors, retinal pigment epithelial cells or RGCs and can be traced to both genetic and environmental factors.^119^^–^^125^

Until recently, it was thought that rod and cone photoreceptor pathways exclusively mediate image-forming functions, where visual acuity and contrast sensitivity were firmly entrenched, and that ipRGCs exclusively mediated non–image-forming functions such as circadian photoentrainment and the PLR. Schmidt et al.^56^ clearly demonstrated that the M4 class of ipRGCs is functionally equivalent to the alpha On RGCs, which are well known for their role in contrast sensitivity. This study showed a functional deficit in contrast sensitivity^56^^,^^87^ in mice lacking M4 ipRGCs, which was further substantiated in mice lacking melanopsin but with the cells intact.^72^ When compared to mice lacking rod or cone photoreceptors, the deficit in contrast sensitivity was greater than the loss in visual acuity, suggesting the relative role of ipRGCs in these different functions and the potential to serve as a biomarker for ipRGC function loss compared to rod and cone function. For context, the OPN4^dta/dta^ mice generally lack all ipRGCs, but the possibility of a few remaining neurons cannot be ruled out.^126^ The OPN4^dta/dta^ mice are presented to illustrate the “floor effect”—that is, the maximal potential effect on outcomes if the ipRGCs were completely degenerated.

 Like visual acuity, contrast sensitivity also decreases with age in humans, starting in the 50s for higher spatial resolution but eventually affecting all spatial resolutions and commencing with mesopic and proceeding to photopic vision loss.^97^^,^^127^ In rodents, contrast sensitivity remains intact up to 18 months old^128^ and decreases by 21 to 24 months old.^129^ Our results are consistent with reported preserved visual acuity in mice up to 12 months old, with the observed small increase in contrast sensitivity at low spatial resolution possibly reflecting compensatory mechanisms or variability in small to medium-sized cohorts. Accelerated loss of contrast sensitivity is a hallmark of AD in humans and a mouse model.^128^

### ipRGCs As a Biomarker in Ocular Diseases

The enduring integrity of ipRGC function throughout adulthood presents a promising biomarker for early neurodegenerative eye diseases. This is particularly relevant considering the widespread loss of ipRGCs in numerous ocular conditions. Aging is one of the main risk factors associated with glaucoma, PD, AD, and diabetes, among others.^30^^–^^44^ Circadian clock disruption also triggers or accelerates the pathology progression in neurodegenerative diseases. For example, in AD, PD, and Huntington's diseases, circadian rhythm alterations seem to trigger or accelerate the pathology progression.^130^^,^^131^ Other authors have reported that alterations of the circadian rhythm include a gradual decrease in nocturnal melatonin secretion^132^ and alterations in sleep.^133^

It has been reported that a loss of circadian rhythms and impairment of pupillary constriction in diseases such as glaucoma, AD, PD, and Huntington's diseases^40^^,^^134^^–^^137^ could also be linked to the ipRGC pathology loss, as well as a loss of the compensatory mechanisms observed in aging. In AD and other neurodegenerative diseases, early circadian rhythm alterations^138^^–^^142^ indicate significant disruptions in the rod and cone photoreceptor pathways, as well as the ipRGC signaling pathways. The reported damage or loss of ipRGCs in the human retina^137^^,^^140^^–^^143^ might account for many related visual dysfunctions, including impaired ocular motility, a reduction in amplitude of the PLR,^135^^,^^136^^,^^143^^,^^144^ and circadian alterations of melatonin.^32^^,^^145^^,^^146^ It is essential to recognize that, while ipRGCs hold promise as potential early biomarkers in certain ocular diseases, their utility might not be consistent across all conditions. Indeed, some diseases have exhibited remarkable resilience in ipRGCs,^147^^–^^150^ showing limited or delayed alterations in these cells despite significant pathological changes occurring elsewhere in the retina. This underscores the complexity of ocular pathophysiology and the need for cautious interpretation when considering ipRGCs as biomarkers. The effectiveness of ipRGCs as diagnostic indicators would likely depend on the specific disease context and the interplay of various underlying factors. As such, the use of ipRGCs as biomarkers demands careful consideration of the unique characteristics of a disease and the role of ipRGCs in the pathogenesis of that disease. To improve our understanding and optimize the diagnostic value of ipRGCs, future research endeavors should focus on exploring the disease-specific roles of ipRGCs and thoroughly investigating the potential limitations they may pose as biomarkers in these early-onset diseases, understanding the dynamics of ipRGC loss to elucidate the specific underlying mechanisms, and assessing their diagnostic and prognostic relevance. More in-depth investigations into the contributions of different ipRGC subtypes, sex-specific differences, and age-related factors are essential to establish ipRGCs as reliable biomarkers for early detection and monitoring of ocular diseases. By gaining a more nuanced understanding of their contributions to various ocular conditions, we can refine their diagnostic applicability and develop tailored approaches for leveraging ipRGCs as valuable tools in the early detection and management of ocular diseases.
